# A bidirectional interfacial engineering strategy for highly stable sodium metal batteries

**DOI:** 10.1039/d5sc04722f

**Published:** 2025-08-26

**Authors:** Xiaomin Yang, Long Wang, Minghui Zhao, Lingxiao Peng, Yun Wu, Baohua Zhu, Le Chen, Jinliang Li

**Affiliations:** a School of Materials Science and Engineering, Guilin University of Electronic Technology Guilin 541004 China 360240512@qq.com; b Siyuan Laboratory, Guangdong Provincial Engineering Technology Research Center of Vacuum Coating Technologies and New Materials, Guangdong Provincial Key Laboratory of Nanophotonic Manipulation, Department of Physics, College of Physics & Optoelectronic Engineering, Jinan University Guangzhou 510632 China lijinliang@email.jnu.edu.cn; c Guangxi Key Laboratory of Optoelectronic Information Processing, School of Optoelectronic Engineering, Guilin University of Electronic Technology Guilin 541004 China chenle11@126.com

## Abstract

Sodium (Na) metal batteries (SMBs) are regarded as some of the most promising next-generation energy storage systems due to their high energy density. However, their practical application is severely hindered by interfacial instabilities at both the anode and cathode, which result in rapid capacity degradation during cycling. Here, we proposed a bidirectional interfacial regulation strategy that simultaneously stabilizes both electrode interfaces. We found that the additive sulfolane features highly polar sulfone groups, effectively tailors the Na^+^ solvation structure and mitigates excessive anion decomposition under high-voltage conditions at the cathode. Concurrently, another additive fluoroethylene carbonate preferentially decomposes at the Na metal anode to form a dense, NaF-rich inorganic layer, which suppresses dendrite growth and inhibits parasitic side reactions. As a result, Na‖Na symmetric cells with this mixed electrolyte exhibit an ultra-long cycling lifespan of 1400 h at 0.5 mA cm^−2^/0.5 mAh cm^−2^, and Na‖Cu cells deliver stable cycling over 500 cycles. Furthermore, the Na‖Na_3_V_2_(PO_4_)_3_ full cell also achieves over 88% capacity retention after 1100 cycles at 80 mA g^−1^. We believe that our work offers a viable pathway for designing high-stability Na metal anodes through synergistic interfacial engineering.

## Abstract 2

We proposed a bidirectional interfacial regulation strategy that simultaneously stabilizes both electrode interfaces in sodium metal batteries.

## Graphical abstract

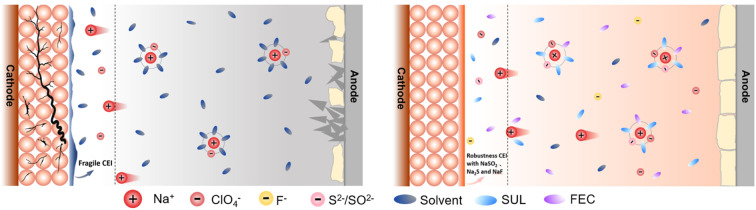

## 1. Introduction

The rapid depletion of fossil fuels and the worsening environmental crisis have accelerated the pursuit of clean and efficient energy storage technologies across multiple disciplines.^1,2^ While lithium-ion batteries (LIBs) have dominated the energy storage field over the past three decades, their large-scale deployment is increasingly constrained by the scarcity of lithium resources and vulnerabilities in global supply chains.^3–5^ As a promising alternative, sodium (Na)-ion batteries (NIBs) have attracted growing attention due to the abundance and low cost of Na.^6,7^ However, conventional NIBs are often limited by low-capacity anodes and high operating voltages, which restrict further improvements in the energy density of NIBs.

To enhance the energy density of NIBs, employing Na metal anodes is considered the most ideal choice, which is due to their high theoretical capacity (1166 mAh g^−1^) and low redox potential (−2.71 V *vs.* SHE).^8^ However, the practical deployment of Na metal anodes is still hindered by severe dendrite growth, resulting in significant safety issues.^9,10^ In addition, parasitic interfacial reactions and volume fluctuations also result in the low utilization efficiency of Na ions, leading to poor cycling stability. To mitigate these issues, current strategies have focused on current collector modification, artificial interfacial layers, and electrolyte engineering.^11–13^ While the former two approaches often suffer from complex fabrication procedures and limited scalability, electrolyte engineering offers high compatibility and facile implementation, and has emerged as the most practical and scalable route.^14^ Nevertheless, conventional liquid electrolytes typically exhibit poor chemical and electrochemical compatibility with Na metal anodes, failing to form stable solid electrolyte interphase (SEI) layers.^15^ This calls for advanced electrolyte designs to stabilize electrode–electrolyte interfaces. Recent studies have demonstrated the potential of functional additives in tailoring SEI chemistry and morphology. Fluoroethylene carbonate (FEC), a representative additive with a low lowest unoccupied molecular orbital (LUMO) energy level, undergoes preferential reduction to form a dense, NaF-rich SEI layer on Na metal surfaces, effectively suppressing dendrite formation and parasitic reactions.^16,17^ However, FEC tends to undergo polymerization under high-voltage conditions, which compromises cathode interfacial stability.^18^ Its decomposition products may also form a thick cathode electrolyte interphase (CEI) layer, impeding ion transport. Overcoming these high-voltage side reactions is still a key challenge in electrolyte design. Fan *et al.* reported the synergistic effect of FEC and SA in enhancing the cycling performance of layered cathodes of NIBs, achieving a capacity retention of 87.2% after 400 cycles at 1C. However, their study did not address the impact of additives on dendrite suppression and the anode interface. In this context, sulfolane (SUL), a high-polarity and highly oxidative-stable solvent, has recently emerged as a promising co-solvent.^19^ Compared to conventional ester solvents such as ethylene carbonate (EC) and propylene carbonate (PC), SUL facilitates the formation of a uniform cathode electrolyte interphase (CEI), thereby enhancing anodic stability and suppressing solvent oxidation at the cathode.^20,21^ Moreover, unlike 1,3,2-dioxathiolane-2,2-dioxide, which primarily adjusts electrolyte viscosity and flowability with limited influence on SEI stabilization, SUL's strong solvation capability offers opportunities to modulate Na^+^ solvation structures at the anode interface.^22^ Nevertheless, SUL alone exhibits limited reductive activity at low potentials, rendering it insufficient for effective SEI formation. Le *et al.* proposed that the combination of SUL and vinylene carbonate (VC) can stabilize PC-based electrolytes and suppress dendrite growth, although the underlying stabilization mechanism was not thoroughly discussed.^23^ While VC enhances electrolyte stability, its performance under high-voltage conditions is inferior to that of FEC. Building on these insights, a dual-additive strategy leveraging the complementary properties of FEC and SUL holds potential to simultaneously stabilize both electrode interfaces; however, this bidirectional interfacial regulation concept remains largely unexplored.

Here, we propose a bidirectional interfacial regulation strategy for SMBs, combining the reductive activity of FEC at the anode with the oxidative stability of SUL at the cathode. FEC preferentially decomposes at the Na metal surface to form an NaF-rich inorganic SEI layer, providing robust electronic insulation and chemical passivation. Simultaneously, SUL enhances cathode interfacial stability by contributing sulfur-containing species to the CEI layer and regulating Na^+^ solvation structures, which facilitate uniform nucleation and accelerate SEI formation at the anode. As a result, the optimized electrolyte achieves an extended electrochemical stability window of 4.9 V and enables long-term cycling stability of 1400 hours in Na‖Na symmetric cells at 0.5 mA cm^−2^/0.5 mAh cm^−2^, as well as 1100 cycles with 88% capacity retention in Na‖Na_3_V_2_(PO_4_)_3_ (NVP) full cells at 80 mA g^−1^. We believe this work provides a practical framework for the rational design of multifunctional electrolytes toward high-energy and long-life SMBs.

## 2. Results and discussion

**Fig. 1:**
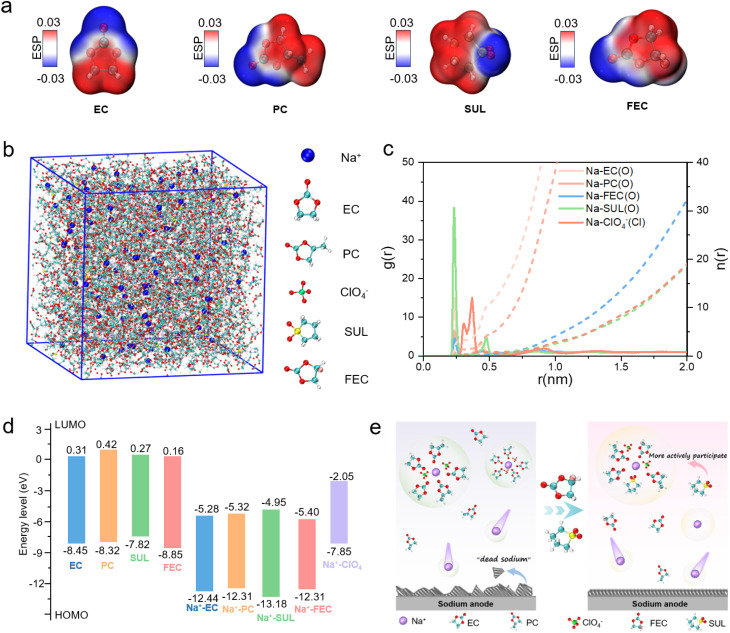
The predicted results by calculation. (a) ESP of EC, PC, FEC and SULs; (b) MD models for EPSF and (c) corresponding RDF curves of EPSF; (d) LUMO and HOMO energy levels of individual molecules and Na^+^ complexes; (e) schematic diagram of the mechanism of electrolytes.

To investigate the influence of various solvents on Na-ion behavior, we calculated the electrostatic potential (ESP) of EC, PC, FEC, and SUL molecules, as shown in Fig. 1a. The results reveal that FEC exhibits a pronounced negative potential region concentrated around the carbonyl group, which is due to the strong electron-withdrawing –F group.^24^ In contrast, SUL displays a distinct negative potential core near the =S=O bond, highlighting the strong polarity of its sulfone group and its ability to strongly coordinate with Na^+^.^25^ To evaluate the solvent effects within electrolytes, we formulated four electrolyte systems: EC/PC (denoted as EP), EC/PC/5% SUL (EPS), EC/PC/5% FEC (EPF), and EC/PC/5% SUL/5% FEC (EPSF) for systematic comparison. Molecular dynamics (MD) simulations were performed to assess the Na^+^ solvation structures in these mixed electrolytes. The MD models for EP, EPS, EPF, and EPSF are presented in Fig. 1b and S1a–c. The corresponding radial distribution functions (RDFs), shown in Fig. 1c and S1d–f, reveal a distinct peak at 0.232 Å upon SUL addition, indicating that SUL molecules enter the first solvation shell of Na^+^.^26^ This interaction weakens the coordination between Na^+^ and ClO_4_^−^ or solvent molecules, thereby suppressing the reductive decomposition of both ClO_4_^−^ and solvents.^27^ This modulation facilitates Na^+^ desolvation, which is beneficial for enhancing battery performance.

To further probe the electrochemical behaviors, we computed the LUMO and highest occupied molecular orbital (HOMO) energy levels of different solvents and their Na^+^-coordinated complexes, as illustrated in Fig. 1d. In general, higher HOMO levels correspond to lower oxidative stability, while lower LUMO levels indicate higher reducibility and greater likelihood of SEI layer formation at the Na metal anode.^28^ Upon Na^+^ coordination, both HOMO and LUMO levels decrease, implying an increased tendency of these complexes to undergo reduction and contribute to SEI formation at the anode, whereas free molecules or ions are more susceptible to oxidation at the cathode interface. Among the solvents, FEC exhibits the lowest LUMO energy, favoring preferential reduction at the Na anode and the formation of a robust SEI layer. In contrast, SUL possesses the highest HOMO level, indicating excellent oxidative stability under high-voltage conditions.^29^ This property extends the electrochemical stability window of the electrolyte and enables the formation of a dense, stable CEI layer on the cathode. Structurally, the SUL molecule features a highly electronegative sulfonyl core (SO_2_), where the S^4+^ center is doubly bonded to oxygen atoms, creating strong negative charge centers.^30^ The cyclic configuration and high dipole moment of the sulfone group impart a high dielectric constant to SUL, which promotes salt dissociation and stabilizes Na-ion transport through the formation of favorable solvation structures. These results indicate a synergistic interfacial regulation mechanism in which FEC predominantly functions at the anode side, facilitating SEI layer formation and suppressing dendritic growth, while SUL contributes to anodic stability under high voltage by mitigating anionic side reactions and suppressing solvent oxidation. This bidirectional modulation significantly enhances the cycling performance of SMBs. Fig. S2 presents the binding energies between the ClO_4_^−^ and different solvents. A significantly more negative binding energy for ClO_4_^−^–FEC can be observed, originating from fluorine substitution-enhanced polarity, reduced steric hindrance, and charge complementarity. Conversely, the least negative binding energy for ClO_4_^−^–SUL is attributed to charge distribution interference from the sulfonyl group, steric hindrance, and high dielectric shielding effects. The synergistic interaction of these factors favorably balances ionic transport, interfacial stability, and redox tolerance within the electrolyte, thereby promoting significant enhancement in SMB performance. Fig. 1e presents the schematic illustration of the dual-function roles of FEC and SUL in interfacial stabilization, and we define this behavior as preferential SEI formation and oxidative resistance.

To validate the solvent structure modulation by the bidirectional interfacial engineering strategy *via* the dual-additive in the electrolyte, we recorded NMR spectra of EP, EPS, EPF, and EPSF. The observed upfield chemical shifts in both the ^1^H (Fig. S3) and ^17^O (Fig. S4) spectra are attributed to the strong electron-withdrawing nature of the two S=O groups in SUL, confirming its influence on the solvation environment.^31,32^ Notably, the Raman spectra (Fig. S5) exhibit a distinct peak at 870 cm^−1^, indicative of a reconstructed ClO_4_^−^ solvation structure triggered by the FEC and SUL additives.^18,33^ Fourier-transform infrared (FTIR) spectroscopy (Fig. S6) confirms the formation of a composite interphase comprising inorganic fluorides (such as NaF), sulfur-containing species (such as RSO_3_Na and Na_2_SO_3_), and organic carbonates or polycarbonates.

**Fig. 2:**
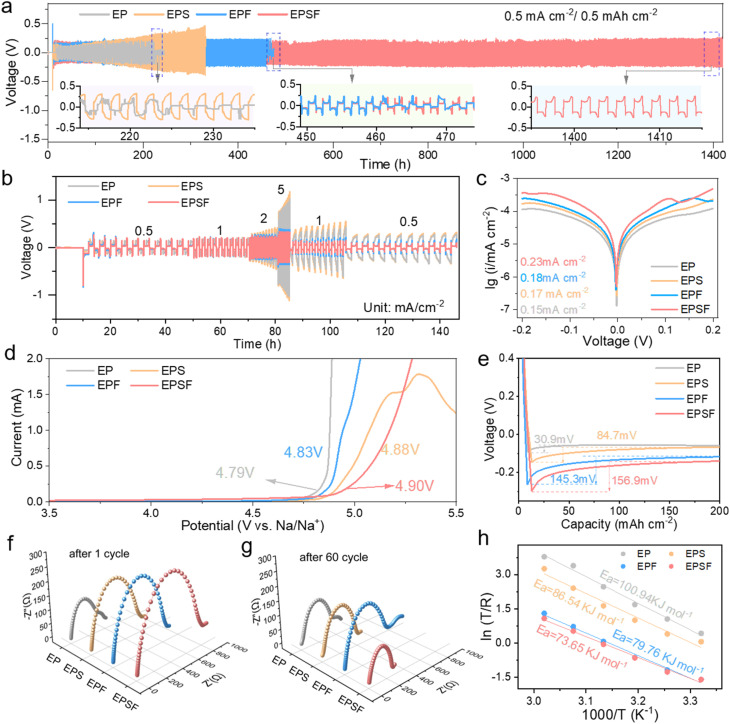
Electrochemical performance of cells with different electrolytes. (a) Cycling and (b) rate performance of Na‖Na cells in EP, EPS, EPF and EPSF electrolytes; (c) Tafel plots, (d) LSV curves and (e) voltage–capacity curve of EP, EPS, EPF and EPSF electrolytes. (f) Nyquist plots of Na‖Na cells after (f) 1 cycle and (g) 60 cycles in EP, EPS, EPF and EPSF electrolytes. (h) Interface desolvation activation energy fitted using the Arrhenius law of EP, EPS, EPF and EPSF electrolytes.

Building upon these molecular-level insights, we explored how the dual-additive strategy influences Na metal anode behavior. We evaluated the electrochemical performance of Na‖Na symmetric cells with EP, EPS, EPF, and EPSF electrolytes at 0.5 mA cm^−2^/0.5 mAh cm^−2^, as shown in Fig. 2a. Cells using the EP electrolyte exhibited short-circuit failure after only 222 h, signifying rapid dendritic growth and separator penetration. The incorporation of SUL (EPS electrolyte) moderately extended the cycling life to 320 h by weakening Na^+^-anion and Na^+^-solvent interactions, facilitating Na^+^ desolvation. However, limited interfacial stability constrained further improvement. With the addition of FEC, the lifespan of the cell with the EPF electrolyte increased to 460 h due to its effective SEI-forming ability at the anode. Strikingly, the dual-additive system (EPSF) delivered a stable cycling time of 1450 h, a marked improvement over all other formulations. To determine the optimal additive ratio, we compared the electrochemical performance of electrolyte systems with different additive concentrations, as shown in Fig. S7. Here, EC/PC with 1% SUL and 1% FEC is defined as 1% EPSF, while EC/PC with 8% SUL and 8% FEC is defined as 8% EPSF. Experimental results reveal that although the 1% EPSF system shows an initial performance improvement, its polarization voltage increases rapidly, indicating that this low concentration cannot sustain long-term interfacial stability. In contrast, the 8% EPSF system retains cycling stability for over 1000 h, but interfacial degradation occurs after 1200 h, ultimately leading to short circuit failure. We attribute this to the fact that excessively high concentrations increase electrolyte viscosity, thereby reducing ionic conductivity. Moreover, the synergistic effect between FEC and SUL at high concentrations may induce excessive reactions or mutual interference, resulting in the deterioration of electrochemical performance. The rate performance under varying current densities (Fig. 2b) revealed that EP and EPS electrolytes led to high overpotentials at elevated current densities, indicating poor Na^+^ transport kinetics and severe interfacial polarization. In contrast, EPF and EPSF electrolytes displayed significantly lower overpotentials, confirming the beneficial interfacial modulation imparted by FEC. Fig. S8 presents the optical microscopy images of Na metal anodes after 60 cycles with different electrolytes. The results further validate the interfacial effects: the EP-based anode exhibited irregular protrusions and pits, whereas the EPSF anode retained a smooth and uniform surface, indicating suppressed dendrite formation.

To further elucidate the interfacial kinetics, Tafel analysis was performed (Fig. 2c). The EP electrolyte exhibited a low exchange current density (*i*_0_) of 0.15 mA cm^−2^, indicating sluggish sodium deposition kinetics, likely due to poor electrode–electrolyte compatibility or inhomogeneous SEI formation.^34^ With the addition of SUL and FEC, the exchange current densities increased to 0.17 mA cm^−2^ (EPS) and 0.18 mA cm^−2^ (EPF), respectively, reflecting enhanced deposition kinetics. Notably, the EPSF electrolyte showed the highest *i*_0_ of 0.23 mA cm^−2^, confirming the synergistic improvement of interfacial kinetics achieved through the dual-additive strategy. Linear sweep voltammetry (LSV) profiles (Fig. 2d) demonstrated that the redox potential increased from 4.79 V (EP) to 4.90 V (EPSF), verifying the extended anodic stability of the dual-additive electrolyte. In addition, we conducted LSV measurements for 1% EPSF and 8% EPSF and compared them with those of EPSF, as shown in Fig. S9. The results show that 8% EPSF exhibits the oxidative decomposition potential of 4.9 V, the same as EPSF. This further confirms that an additive concentration of 5% represents the critical point for performance optimization, and increasing the concentration beyond this level does not enhance electrochemical stability. We also evaluated the nucleation overpotential of Na plating, as shown in Fig. 2e. Compared to EP (30.9 mV), EPS (84.7 mV), and EPF (145.3 mV), the EPSF system exhibited the highest nucleation overpotential of 156.9 mV. This elevated energy barrier promotes homogeneous nucleation at multiple active sites, leading to uniform Na deposition and reduced dendrite growth.^35^ Electrochemical impedance spectroscopy (EIS) was further used to evaluate the interfacial resistance of Na‖Na cells before and after cycling, as shown in Fig. 2f. Initially, EP exhibited the lowest impedance due to its low initial interfacial resistance, while EPSF showed higher resistance attributed to the sluggish Na^+^ transport kinetics of FEC and SUL. However, the impedance of EP increased significantly after 60 cycles, which is due to thick and poorly conductive SEI formation.^36^ whereas the resistance of all additive-containing electrolytes decreased, indicating stable and conductive interphase formation. The lowest interfacial resistance observed with EPSF after cycling confirms the formation of a compact and protective SEI layer, capable of effectively suppressing dendrite growth and mitigating side reactions. As shown in Fig. 2h, the EP electrolyte exhibits the highest desolvation energy (100.94 kJ mol^−1^), indicating a substantial energy barrier during the desolvation process. The introduction of the SUL additive reduces the activation energy (*E*_a_) to 86.54 kJ mol^−1^, highlighting its effectiveness in lowering desolvation barriers and enhancing interfacial dynamics. The EPSF electrolyte shows the lowest desolvation energy, reflecting the synergistic regulation of FEC and SUL. We also performed MD simulations to calculate the mean squared displacement (MSD) of Na^+^ in different electrolyte systems (Fig. S10). The EPSF electrolyte exhibits a *D*_Na^+^_ of 9.63 × 10^−8^ cm^2^ s^−1^, which is markedly higher than that of the other three electrolytes, further demonstrating the pronounced promoting effect of SUL introduction on the kinetic performance.

**Fig. 3:**
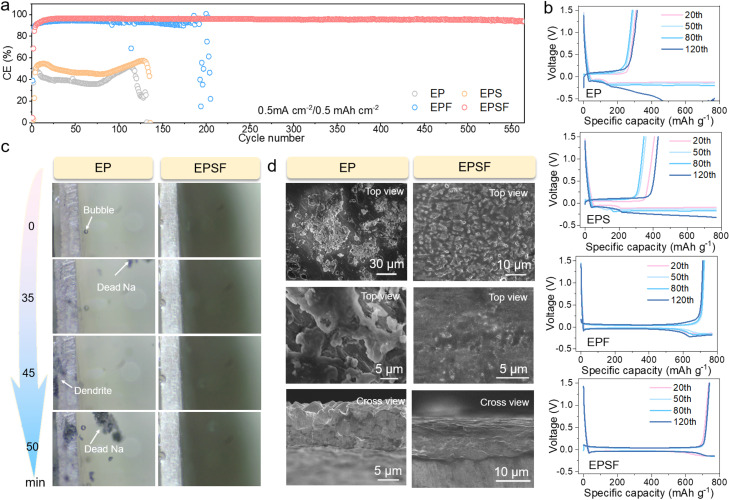
Electrochemical performance of Na‖Cu cells and interface evolution of the Na metal anode. (a) CE and (b) GCD curves of Na‖Cu cells with different electrolytes. (c) *In situ* optical microscopy of the electrode–electrolyte interface on the Na anode with EP and EPSF electrolytes. (d) SEM images of Cu anodes of Na‖Cu cells after the 50th cycle.

To evaluate the reversibility, we further investigated the coulombic efficiency (CE) of Na‖Cu asymmetric cells using various electrolytes at 0.5 mA cm^−2^/0.5 mAh cm^−2^, as shown in Fig. 3a. In EP and EPS electrolytes, the CE remained below 70% and exhibited a marked decline after approximately 120 cycles, indicative of irreversible parasitic reactions or Na loss during plating/stripping. In contrast, the incorporation of FEC significantly improved Na reversibility, enabling the EPF-based cell to sustain cycling up to 173 cycles before a pronounced CE drop was observed. Notably, the dual-additive electrolyte (EPSF) delivered a substantial enhancement in reversibility, supporting stable cycling over 550 cycles, which confirms the synergistic effect of FEC and SUL in mitigating heterogeneous Na deposition and suppressing side reactions. Fig. 3b shows the galvanostatic charge/discharge (GCD) profiles of Na‖Cu cells. The results further reveal that cells using EP or EPS electrolytes suffer from large polarization voltages between plating/stripping processes, suggesting sluggish interfacial kinetics associated with unstable SEI formation. By contrast, EPF and EPSF electrolytes exhibit reduced voltage hysteresis, demonstrating enhanced interfacial kinetics attributed to the formation of a more uniform and stable SEI layer.^37^ To gain deeper insights into the interfacial behavior, we employed *in situ* optical microscopy to visualize the dynamic evolution of Na deposition in EP and EPSF electrolytes (Fig. 3c). It is found that rapid Na dendritic growth can be observed and the emergence of “dead Na” became apparent after 35 min in EP, likely caused by bubble-induced detachment of loosely deposited dendrites. Conversely, the Na surface remained smooth and compact in EPSF, with no evident dendrite formation even after 50 min Na plating. The corresponding videos (Videos S1 and S2) display the real-time Na deposition behavior in Na‖Na cells, further validating the additive-enabled dendrite suppression. Furthermore, SEM analyses of Cu electrodes retrieved from Na‖Cu asymmetric cells after 100 h cycling at 0.5 mA cm^−2^ (Fig. 3d) reveal a striking contrast in Na deposition morphology. In EP, surface irregularities such as fine ridges and protrusions promote localized Na accumulation, resulting in heterogeneous plating and “dead Na” formation, as evidenced by a rough surface with extensive dendritic structures.^38^ In contrast, EPSF facilitates smooth, dendrite-free Na deposition, with the Cu surface remaining flat and uniform, underscoring the effectiveness of the dual-additive strategy in regulating interfacial Na behavior. Therefore, we believe that this co-functionalization by FEC and SUL synergistically strengthens SEI layer mechanical integrity and interfacial kinetics, substantially suppressing dendritic growth.^39^

**Fig. 4:**
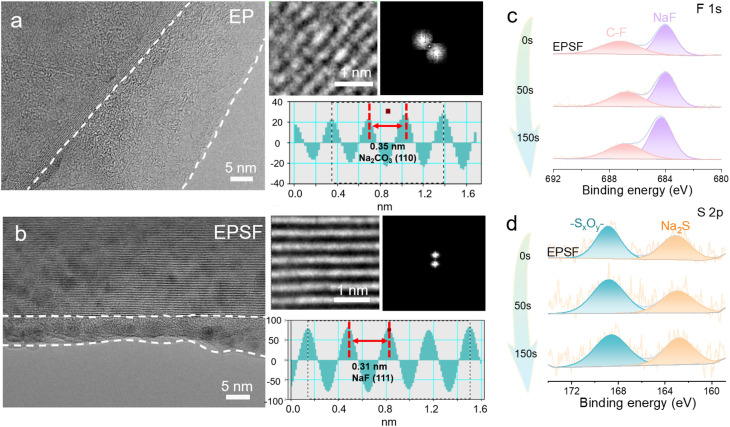
Interface characteristics of the Na metal anodes with electrolytes. High-resolution cryo-TEM images of the SEI layer in (a) EP and (b) EPSF electrolyte. Insets show the fast Fourier transform patterns and line intensity profiles. (c) F 1s and (d) S 2p XPS spectra of the SEI layer on Na after 10 cycles with the EPSF electrolyte.

To further investigate the influence of electrolytes on the Na metal anode, we conducted post-mortem analyses of the SEI layer after cycles. Fig. 4 presents the cryogenic transmission electron microscopy (cryo-TEM) images of the residual deposits on the Cu surface. As shown in Fig. 4a, the SEI formed in the EP electrolyte exhibits a thickness of ∼25 nm. High-resolution imaging reveals that the majority of this interfacial layer is amorphous, consistent with its predominantly organic composition. Within this region, some nanocrystalline domains are observed, and further lattice-resolved analysis confirms the presence of Na_2_CO_3_. The lattice spacings correspond well with Na_2_CO_3_ crystallography, which likely originates from the decomposition of EP-based solvents and subsequent generation of CO_2_.^40^ In contrast, cryo-TEM imaging of the SEI layer formed in the EPSF electrolyte reveals a significantly thinner interfacial layer of 7 nm (Fig. 4b). A thinner SEI generally implies reduced interfacial side reactions and a more stable electrode–electrolyte interface, leading to higher CE and enhanced cycling stability.^41^ Magnified views of the EPSF-derived SEI layer exhibit well-defined lattice fringes, which are indexed to crystalline NaF. These findings confirm that the co-addition of FEC and SUL promotes the formation of a NaF-rich SEI layer, primarily resulting from the reductive decomposition of FEC. The presence of such fluorinated species enhances SEI robustness, suppresses dendritic Na growth, and contributes to improved cycling performance. The SEI composition and structure resulting from the EPSF electrolyte were examined using X-ray photoelectron spectroscopy (XPS), as shown in Fig. 4c and d. The F 1s spectra reveal that FEC preferentially decomposes on the sodium metal anode to generate a NaF-rich inorganic layer. Furthermore, the detection of –S_*x*_O_*y*_– and Na_2_S species indicates decomposition products of the SUL additive, corroborating its participation in forming the inner Na^+^ solvation shell and regulating the Na^+^ solvation structure. Overall, the EPSF electrolyte is shown to yield a more stable, less reactive, and mechanically robust SEI on the sodium metal surface, in agreement with the cryo-TEM observations.

**Fig. 5:**
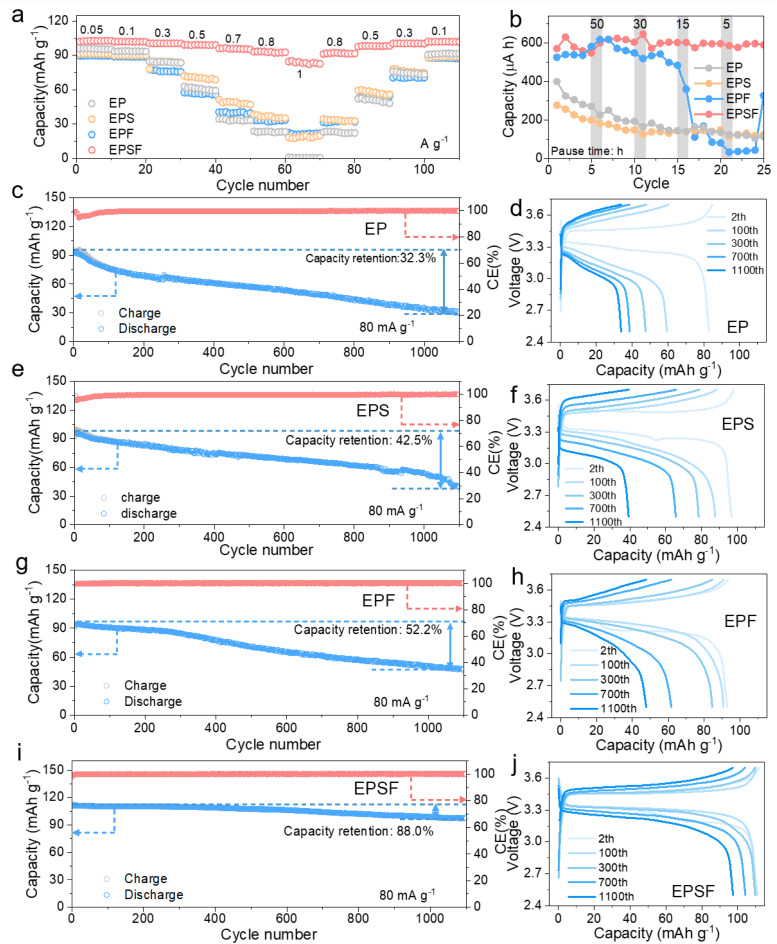
Electrochemical performance of the Na‖NVP full cell. (a) Rate performance and (b) SEI dissolution analyses based on capacity monitoring of the cells with EP, EPF, PES, and EPSF electrolytes. Cycling performance of the Na‖NVP full cell in (c) EP, (d) EPF, (e) PES, and (f) EPSF electrolytes and (g–j) the corresponding GCD curves.

To evaluate the practical applicability, we assembled Na‖NVP cells to further assess the electrochemical performance with different electrolytes. Fig. 5a and S11 present the rate and corresponding CE performance with different electrolytes. It is found that the cell with the EP electrolyte exhibited a rapid capacity decline as the current density increased, with the capacity nearly dropping to 1 mAh g^−1^ at 1 A g^−1^. The incorporation of a single additive resulted in marginal improvement. However, the full cell employing the EPSF electrolyte demonstrated a remarkable enhancement of the rate performance, with the specific capacities of 102.7, 101.8, 100.4, 99.0, 95.0, 92.6, and 82.5 mAh g^−1^ at 50, 100, 300, 500, 700, 800, and 1000 mA g^−1^, respectively. This significant improvement can be attributed to the synergistic effects of FEC and SUL, which jointly optimize the electrode–electrolyte interface, reduce interfacial resistance, and facilitate rapid Na^+^ transport. Fig. 5b presents dissolution analyses based on capacity monitoring of Na‖Cu cells using EP, EPF, PES, and EPSF electrolytes after resting for various intervals (50, 30, 15, and 5 h) following every 5 cycles at 0.5 mA cm^−2^. The addition of FEC supplies a sufficient amount of NaF, effectively reducing SEI dissolution. Meanwhile, SUL modulates the Na^+^ solvation structure, enhancing Na^+^ mobility and further mitigating SEI layer dissolution and uneven deposition. Through the synergistic effect of SUL and FEC, the EPSF electrolyte forms a more stable, less reactive, and mechanically robust SEI on the sodium metal anode, enabling it to retain higher capacity with slower decay over multiple cycles. Notably, the slight capacity increase following the rest periods is attributed to the replenishment of the CEI *via* dissolution under open-circuit conditions. We further investigated the cycling performance of Na‖NVP full cells with different electrolytes at 80 mA g^−1^, as shown in Fig. 5c–j. The cell with the EP electrolyte showed a capacity retention of only 32.3% after 1150 cycles. In comparison, cells with EPS and EPF electrolytes achieved higher capacity retentions of 42.5% and 52.2% under the same conditions, respectively. Remarkably, with the dual-additive EPSF electrolyte, the cell retained a high specific capacity of 97.8 mAh g^−1^ after 1150 cycles with a capacity retention of 88.0%. This clearly demonstrates the superior stabilizing effect of the dual-additive strategy on the long-term cycling performance.

**Fig. 6:**
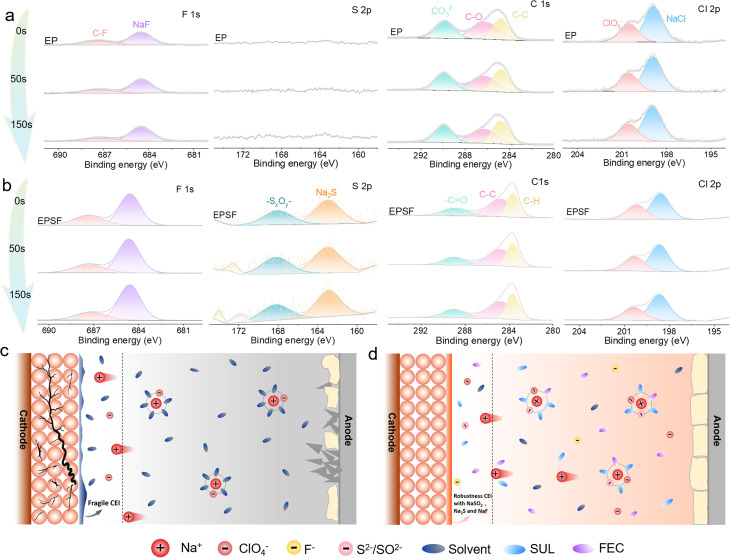
Interface characteristics of the cathode with electrolytes and the schematic of the bidirectional interfacial engineering strategy. F 1s, S 2s, C 1s, and Cl 2p XPS spectra of the NVP cathodes with (a) EP and (b) EPSF electrolytes after 180 cycles. Schematic of the cathode stabilizing mechanism in (c) EP and (d) EPSF electrolytes.

To investigate the mechanism underlying the enhanced cathode stability enabled by the dual-additive strategy, we provide the SEM images to analyze the NVP cathode after 180 cycles in both EP and EPSF electrolytes, as shown in Fig. S12. Pronounced surface cracks and particle fragmentation were observed on the cathode cycled in EP, corroborating the aforementioned severe degradation. To further probe the interfacial chemistry, XPS was conducted on the cathode surface after 200 cycles to identify the composition of the CEI, as shown in Fig. 6a and b. The CEI formed in EP was predominantly composed of organic species, primarily originating from solvent decomposition.^42,43^ In contrast, the CEI derived from EPSF showed an increased proportion of fluorinated species, indicating a higher NaF content. The enrichment of NaF plays a crucial role in passivating the cathode surface, stabilizing the electrode–electrolyte interface, and preventing excessive oxidative decomposition of solvents and salts. With the introduction of the SUL additive, sulfur-rich inorganic components were also detected in the CEI, which contribute to improved physical robustness and enhanced interfacial stability. Fig. S13 shows the percentage statistics of C–F and NaF components at different depths in the corresponding CEI layer with EP and EPSF electrolytes, which also confirm this result. In the C 1s spectrum, the CEI derived from EP shows peaks corresponding to CO_3_^2−^, C–O, and C–C species, suggesting extensive decomposition of EC and PC solvents into carbonates and partially oxidized organics. The presence of CO_3_^2−^ is a typical indicator of electrolyte degradation at the cathode, which correlates with capacity fading. In contrast, the CEI layer formed in the EPSF electrolyte exhibited peaks assigned to more stable –C=O and C–H groups, suggesting the formation of a protective interphase that suppresses solvent decomposition due to the coordinated action of FEC and SUL. The Cl 2p spectra revealed negligible change in the decomposition products of ClO_4_^−^, indicating that NaClO_4_ remained largely intact and was not significantly decomposed. This further confirms that FEC and SUL effectively stabilize the CEI layer and inhibit overall electrolyte degradation. Considering the additive roles in CEI formation, FEC likely undergoes preferential decomposition at the cathode surface, forming a more stable CEI layer that protects against further EC/PC breakdown and thereby suppresses CO_3_^2−^ generation. Meanwhile, the high oxidative stability of the sulfone-based SUL reduces electrolyte decomposition at high voltages, minimizing oxidative by-products (Fig. 6c and d). Overall, the dual-additive strategy enables the construction of a flexible rigid composite CEI layer, which enhances interfacial stability and improves Na^+^ transport kinetics.

## 3. Conclusion

In summary, we proposed a bidirectional interfacial regulation strategy that simultaneously stabilizes both electrode interfaces. We found that the additive SUL features highly polar sulfone groups, which effectively tailors the Na^+^ solvation structure and mitigates excessive anion decomposition under high-voltage conditions at the cathode. Concurrently, another additive FEC preferentially decomposes at the Na metal anode to form a dense, NaF-rich inorganic layer, which suppresses dendrite growth and inhibits parasitic side reactions. As a result, Na‖Na symmetric cells with this mixed electrolyte exhibit an ultra-long cycling lifespan of 1400 h at 0.5 mA cm^−2^/0.5 mAh cm^−2^, and Na‖Cu cells deliver stable cycling over 500 cycles. Furthermore, the Na‖NVP full cell also achieves over 88% capacity retention after 1100 cycles at 80 mA g^−1^. We believe that our work offers a viable pathway for designing high-stability Na metal anodes through synergistic interfacial engineering.

## Author contributions

X. Yang performed investigation, methodology, data curation, wrote the original draft. L. Wang, M. Zhao, L. Peng, Y. Wu performed investigation. B. Zhu, L. Chen, and J. Li performed supervision, conceptualization and wrote, review & edited the final manuscript.

## Conflicts of interest

The authors declare no conflict of interest.

## Supplementary Material

SC-016-D5SC04722F-s001

SC-016-D5SC04722F-s002

SC-016-D5SC04722F-s003

## Data Availability

The data supporting this article have been included as part of the SI. See DOI: https://doi.org/10.1039/d5sc04722f.
