# Supplementary material for: A bidirectional interfacial engineering strategy for highly stable sodium metal batteries
Source: Chem Sci. 2025 Aug 26;16(38):17703–13. doi: 10.1039/d5sc04722f (PMC12406151; doi:10.1039/d5sc04722f)
Supplement: SC-016-D5SC04722F-s001 [file SC-016-D5SC04722F-s001.pdf]

Supplementary Information (SI) for Chemical Science.
This journal is © The Royal Society of Chemistry 2025

Supporting Information

Bidirectional interfacial engineering strategy for highly stable sodium metal batteries

Xiaomin Yang[a], Long Wang[b], Minghui Zhao[a], Lingxiao Peng[c], Yun Wu[c], Baohua Zhu*[a], Le Chen*[c], Jinliang Li*[b]

[a]*School of Materials Science and Engineering, Guilin University of Electronic Technology, Guilin 541004, China, Email: 360240512@qq.com*

[b]*Siyuan Laboratory, Guangdong Provincial Engineering Technology Research Center of Vacuum Coating Technologies and New Materials, Guangdong Provincial Key Laboratory of Nanophotonic Manipulation, Department of Physics, Jinan University, Guangzhou 510632, China. Email: lijinliang@email.jnu.edu.cn*

[c]*Guangxi Key Laboratory of Optoelectronic Information Processing, School of Optoelectronic Engineering, Guilin University of Electronic Technology, Guilin 541004, China. Email: chenle11@126.com*

Experimental

Electrolyte

The EP (baseline) electrolyte was prepared by dissolving 1 mol L^{-1} $NaClO_4$ in a solvent mixture of EC and PC with a volume ratio of 1:1. To obtain the EPSF electrolyte, 5 wt% FEC and 5 wt% SUL were simultaneously added to the baseline electrolyte. For comparison, 5 wt% SUL or 5 wt% FEC was individually added to the baseline electrolyte to prepare EPS and EPF, respectively. All electrolyte preparations and storage were conducted in an argon-filled glovebox (O_2 < 0.1 ppm, H_2O < 0.1 ppm). Unless otherwise specified, all electrolytes were allowed to stand for 24 h prior to use.

Characterization

The morphologies of the Na metal anode, SEI layer and NVP electrode were obtained by optical microscope (Leica), TEM (TEOL), and SEM (FEI). The Raman spectrometer (WITec), and XPS (Thermo Scientific). For the in-situ visualization observation, we used a portable optical microscope and cooperated with an electrochemical reaction cell to realize the in situ observation of the electrode.

Electrochemical performance

The electrochemical performances of all the cells with different electrolytes were tested with CR2032 coin-type cells, which were assembled in an argon-filled (O_2<0.1 ppm, H_2O<0.1 ppm) glove box (Etelus Lab2000). The symmetric cell was assembled by the Na metal electrode at both ends of the cell. The cells of Na//Al, Na//Cu and Na//NVP was assembled with one piece of Na foil as the counter electrode and a work electrode of Al, Cu and NVP, respectively, with glass fiber as the separator. The cycling performance of GCD curves were tested by the Neware battery test system (Neware BTS-4000). EIS and nucleation overpotential of the symmetric cells were recorded by Chenhua electrochemical workstation. The activation energy for de-solvation (E_{ct}) during Na deposition process was calculated by Arrhenius equation:

$$\frac{T}{R_{ct}} = Aexp\left(\frac{E_{ct}}{RT}\right)$$

where R, T, A, and R_{ct} are the standard gas constant, temperature, pre-exponential factor, and the fitting resistance of symmetric cells at different temperatures.

Calculations

MD simulations were conducted with GROMACS software. A time step of 1 fs was employed, with a cutoff distance of 1.0 nm for both the van der Waals interactions and electrostatic interactions. The electrostatic interactions were calculated by the particle mesh Ewald method (PME). In the NVT simulations, the V-rescale thermostat with a characteristic time of 2 ns was applied to implemented the constant temperature condition. For the NPT simulations, the pressure was maintained at 1 atm via an isotropic Parrinello Rahman barostat. The size of the cubic box is 10 x 10 x 10 nm^3 (with periodic boundary conditions). System 1 contains 100 Na^+,100 ClO_4^-, 738 EC and 588 PC. System 2 contains 100 Na^+,100 ClO_4^-,738 EC 588 PC and 52 SUL. System 3 contains 100 Na^+,100 ClO_4^-,738 EC, 588 PC and 59 FEC. System 4 contains 100 Na^+,100 ClO_4^-,738 EC, 588 PC, 59 FEC and 52 SUL. The interaction parameters of various ions and organic molecules were taken from GAFF force field. The MD simulations were carried out for 30 ns to ensure the system reaching equilibrium, and the last 5 ns of trajectories were used for post-processing analysis.

The quantum chemistry calculations were conducted by Gaussian (G09) software. The geometry optimization and the single point energy calculations were employed at B3LYP/def2svp and B3LYP/def2tzvp level, respectively. The iso-surfaces of ESP were displayed by VMD package.

Table S1. Electrostatic, Van der Waals and Total interactions in EP electrolyte.

EP	Electrostatic interaction /kJ mol^{-1}	Van der Waals interaction /kJ mol^{-1}	Total Interaction (Summation) /kJ mol^{-1}
Na-EC	-22860.2	2071.84	-20788.36
Na-PC	-7811.49	582.974	-7228.516

Table S2 Electrostatic, Van der Waals and Total interactions in EPS electrolyte.

EPS	Electrostatic interaction /kJ mol^{-1}	Van der Waals interaction /kJ mol^{-1}	Total Interaction (Summation) /kJ mol^{-1}
Na-EC	-21717.1	2058.63	-19658.47
Na-PC	-7246.89	569.147	-6677.743
Na-SUL	-2503.37	228.686	-2274.684

Table S3 Electrostatic, Van der Waals and Total interactions in EPF electrolyte.

EPF	Electrostatic interaction /kJ mol^{-1}	Van der Waals interaction /kJ mol^{-1}	Total Interaction (Summation) /kJ mol^{-1}
Na-EC	-21947.1	2067.67	-19879.43
Na-PC	-7775.48	611.398	-7164.082
Na-FEC	-554.79	40.5553	-514.2347

Table S4 Electrostatic, Van der Waals and Total interactions in EPSF electrolyte.

EPSF	Electrostatic interaction /kJ mol^{-1}	Van der Waals interaction /kJ mol^{-1}	Total Interaction (Summation) /kJ mol^{-1}
Na-EC	-20676.9	1971.22	-18705.68
Na-PC	-7638.18	600.877	-7037.303
Na-FEC	-525.33	40.0147	-485.3153
Na-SUL	-2811.33	258.01	-2553.32

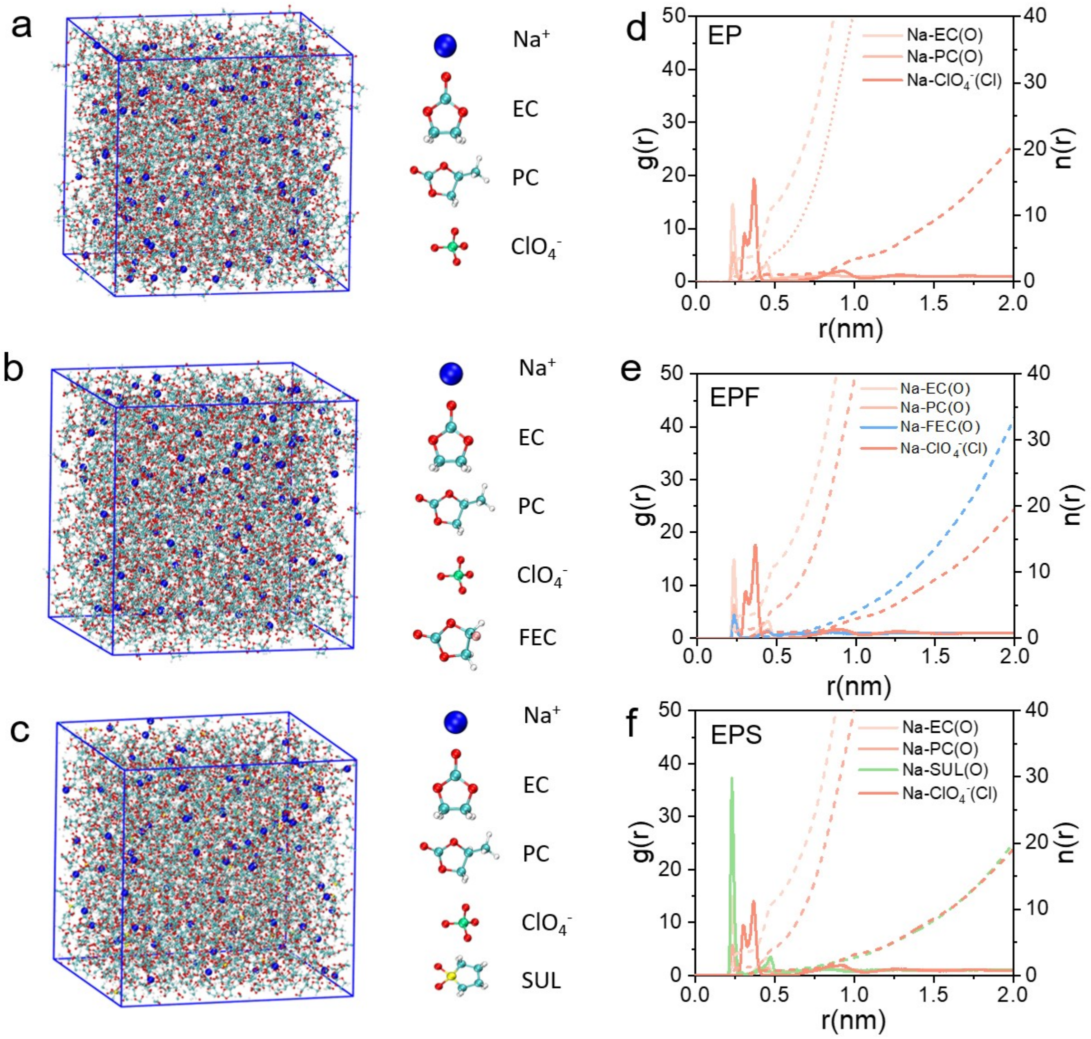

Figure S1 MD models for (a) EP, (b) EPF, (c) EPS and corresponding RDF curves of (d) EP, (e) EPF, (f) EPS.

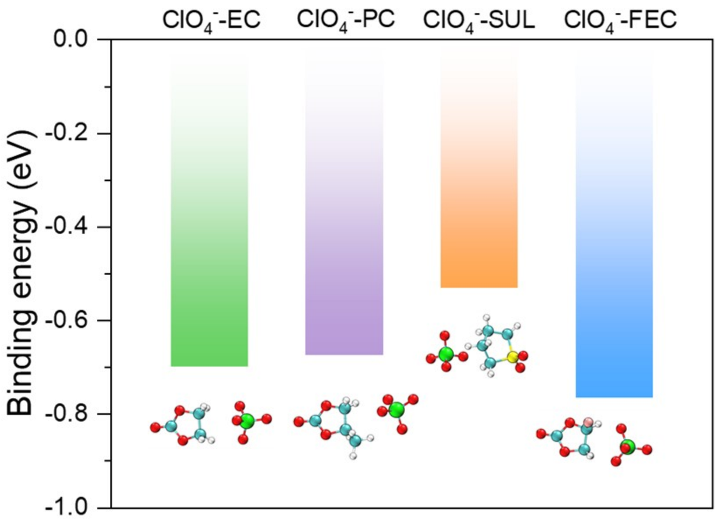

Figure S2 Binding energy of ClO_4^- with solvent molecules.

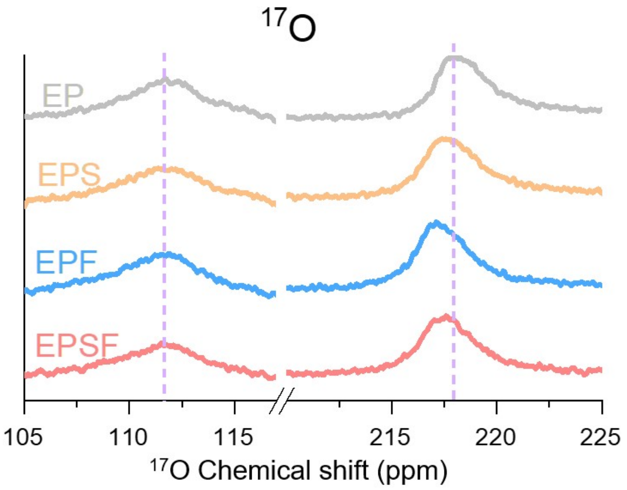

Figure S3 ^{17}O NMR spectra of EP, EPS, EPF and EPSF electrolytes.

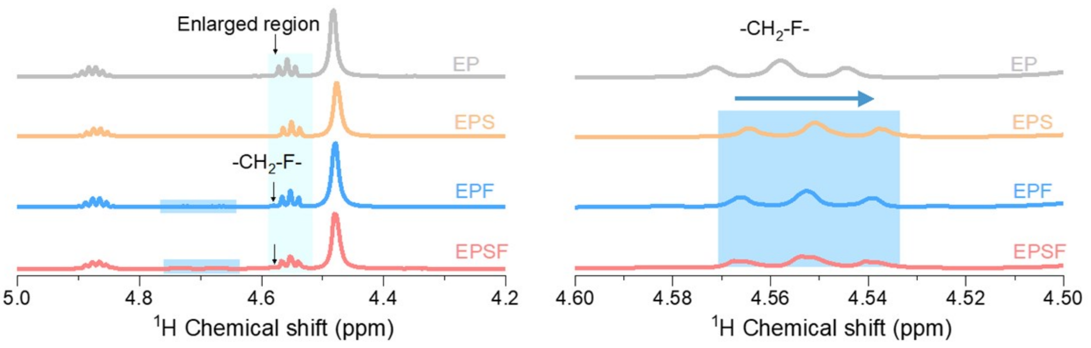

Figure S4 ^{1}H NMR and the enlarge NMR spectra of EP, EPS, EPF and EPSF electrolytes.

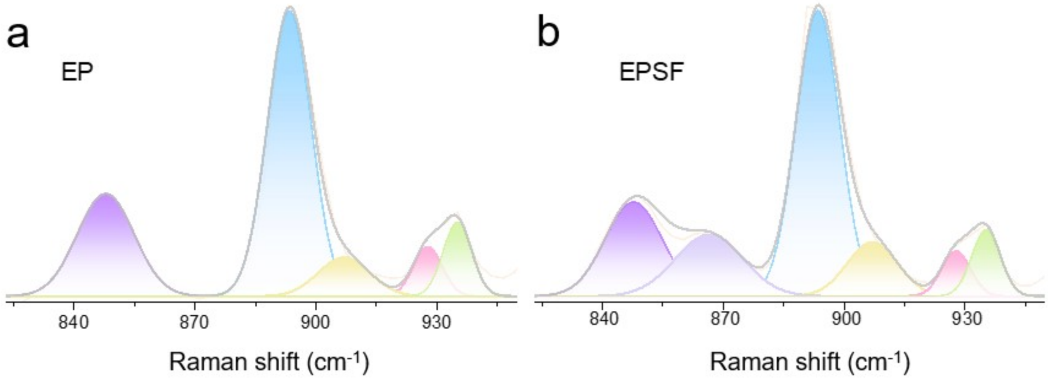

Figure S5 Raman spectra of (a) EP and (b) EPSF electrolytes.

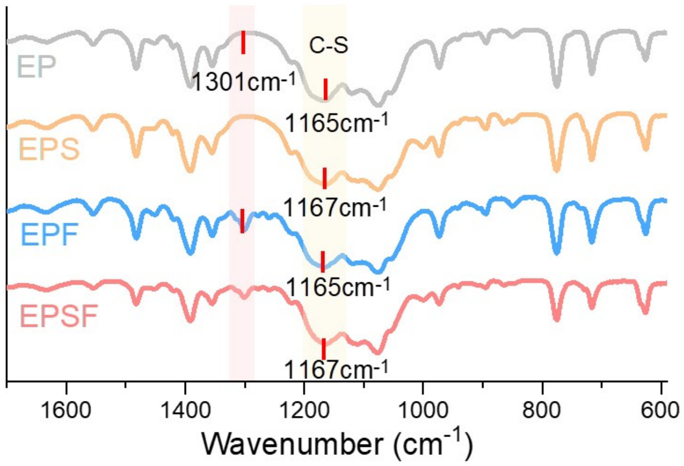

Figure S6 FTIR spectra of EP, EPS, EPF and EPSF electrolytes.

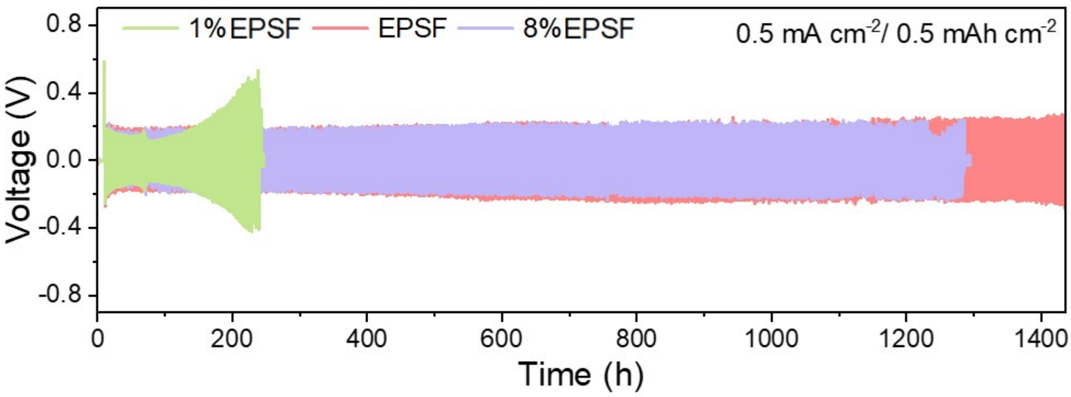

Figure S7 Long-term cycling of Na symmetric cell in 1% EPSF ,5% EPSF and 8%EPSF electrolytes;

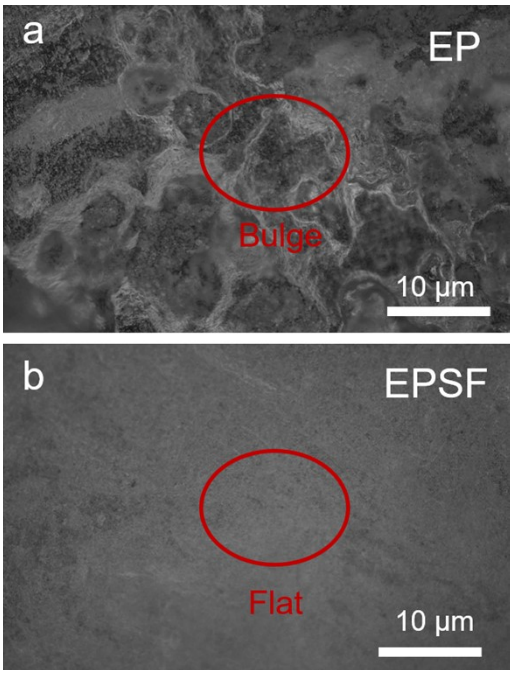

Figure S8 Optical microscopy of Na metal anode with EP and EPSF electrolytes after 60 cycles.

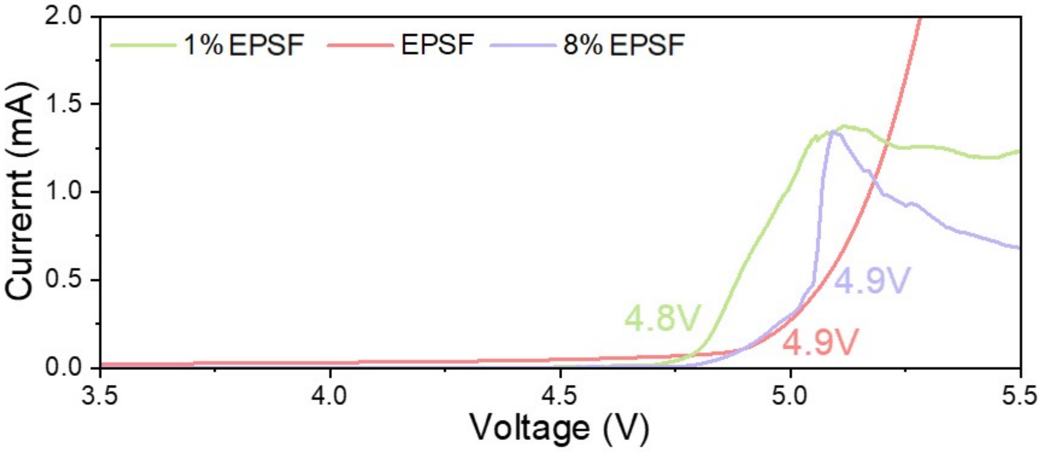

Figure S9 LSV curves of 1% EPSF, EPSF and 8% EPSF electrolytes.

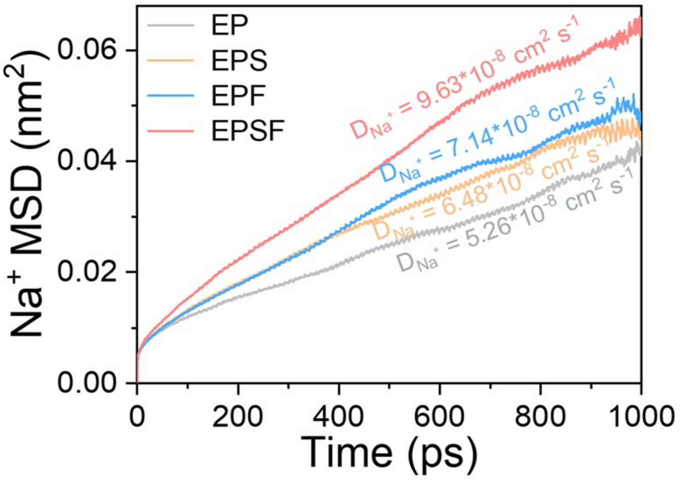

Figure S10 Na^+ MSD vs Time for Different Electrolyte Systems

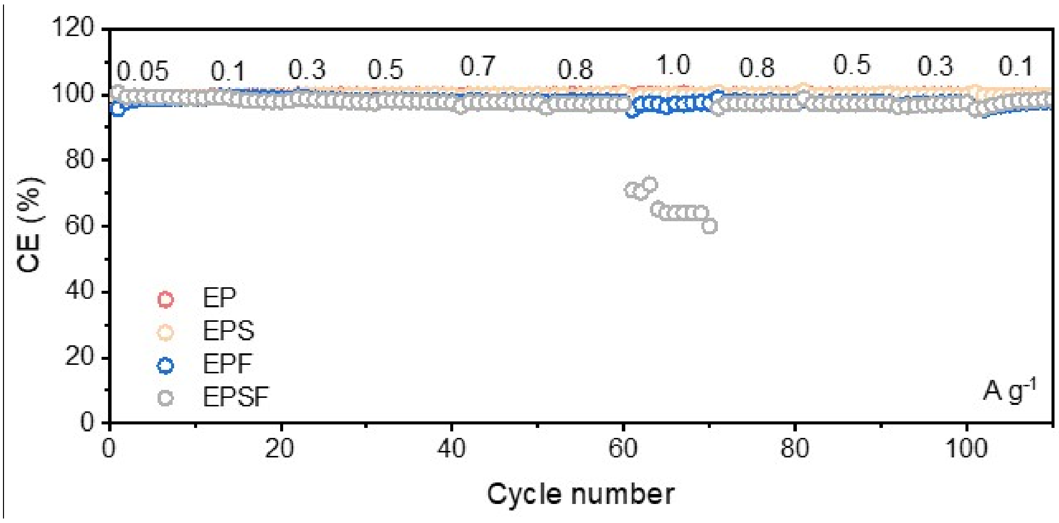

Figure S11 CE of Na||NVP full cell with EP, EPF, PES, and EPSF electrolytes at different current densities.

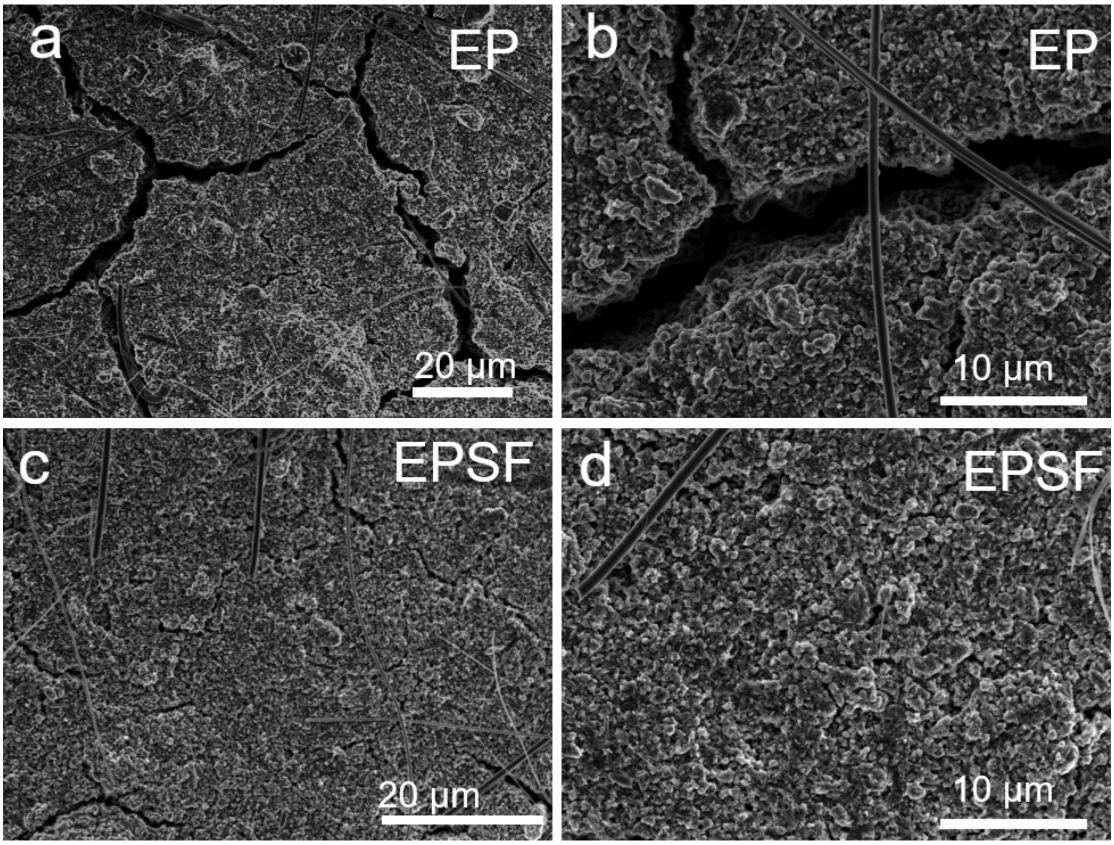

Figure S12 SEM images of NVP electrodes with (a, b) EP and (c, d) EPSF electrolytes

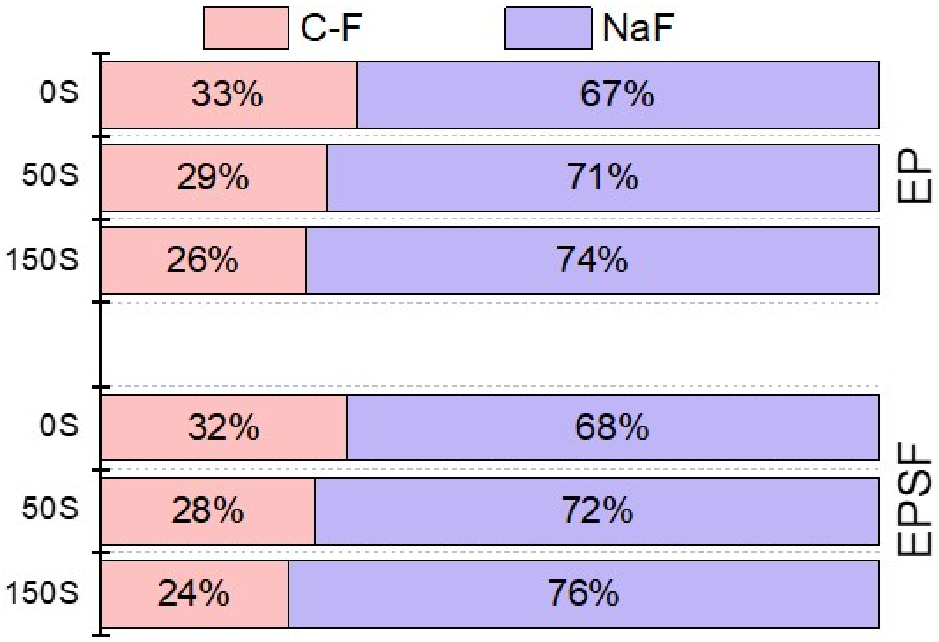

Figure S13 Percentage statistics of C-F and NaF components at diferent depths in the corresponding CEI.
